# 173. Deciphering COVID-19-Associated Effects on Hospital MRSA Transmission and Social Networks

**DOI:** 10.1093/ofid/ofab466.173

**Published:** 2021-12-04

**Authors:** Gregory Madden, Matthew Bielskas, Methun Kamruzzaman, Parantapa Bhattacharya, Bryan Lewis, Eili Klein, Costi Sifri, Anil Vullikanti

**Affiliations:** 1 Division of Infectious Diseases & International Health, Charlottesville, VA; 2 UVA Biocomplexity Institute, Charlottesville, Virginia; 3 Center for Disease Dynamics, Economics & Policy, Silver Sping, Maryland; 4 Office of Hospital Epidemiology/Infection Prevention & Control, UVA Health, Charlottesville, VA, Charlottesville, Virginia

## Abstract

**Background:**

The COVID-19 pandemic was associated with a significant (28%) reduction of methicillin-resistant *Staphylococcus aureus* (MRSA) acquisition at UVA Hospital (*P*=0.016). This “natural experiment” allowed us to analyze 3 key mechanisms by which the pandemic may have influenced nosocomial transmission: 1) enhanced infection control measures (i.e., barrier precautions and hand hygiene), 2) patient-level risk factors, and 3) networks of healthcare personnel (HCP)-mediated contacts.

Figure 1. Monthly MRSA Acquisition Rates Pre- and Post-COVD-19

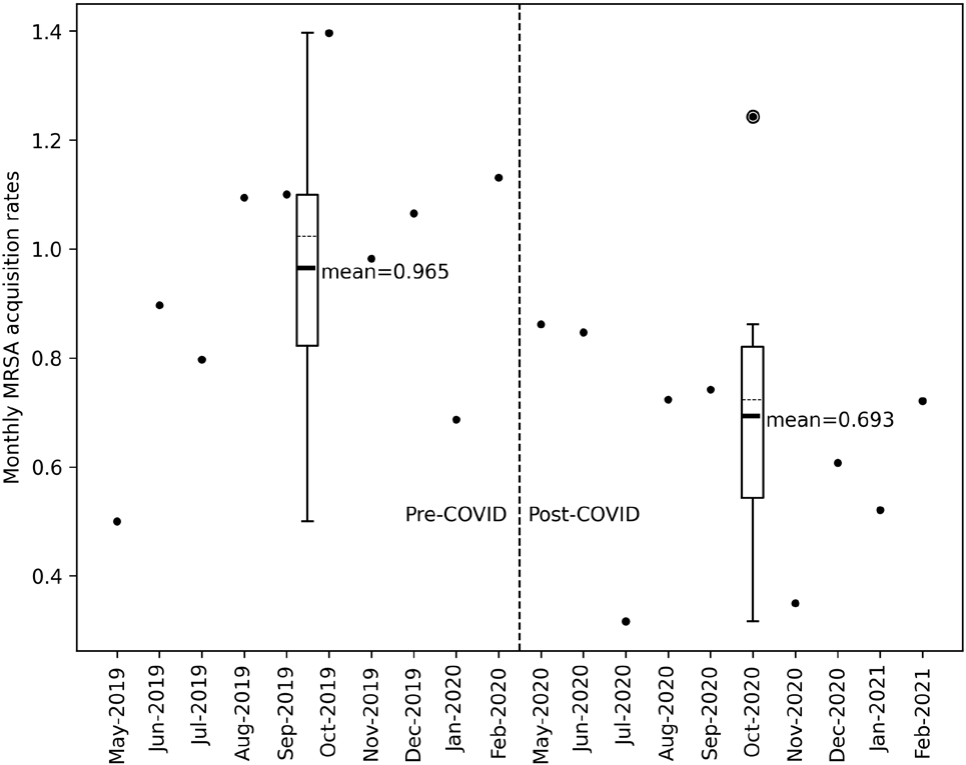

Hospital MRSA acquisition was defined as a new clinical or surveillance positive in patients with prior unknown or negative MRSA status occurring >72h after admission. 10 month time periods pre- (5/6/2019 to 2/23/2020) and post-COVID-19 (5/4/2020 to 2/28/2021) were chosen to mitigate the effects of seasonality. A 6-week wash-in period was utilized coinciding with the onset of several major hospital-wide infection control measures (opening of 2 special pathogen units with universal contact/airborne precautions on 4/1/21 and 5/1/21, universal mask 4/10/21 and eye protection 4/20/20 policies instituted along with staff education efforts including the importance of standard precautions). Box and whisker plots depict quartile ranges, median (dotted line), and mean values. Mean MRSA acquisition rates pre- (0.92 events per 1,000 patient days) significantly declined post-COVD-19 (to 0.66; P=0.016). Independent-samples t tests were used (2-tailed) for statistical comparisons except for variables without a normal distribution (Shorr Scores), for which a Mann-Whitney U test was used.

**Methods:**

Census-adjusted hospital-acquired MRSA acquisition events were analyzed over 10 months pre- (5/6/2019 to 2/23/2020) and post-COVD-19 (5/4/2020 to 2/28/2021), with a 6-week wash-in period coinciding with hospital-wide intensification of infection control measures (e.g., universal masking). HCP hand hygiene compliance rates were examined to reflect adherence to infection control practices. To examine impacts of non-infection control measures on MRSA transmission, we analyzed pre/post-COVD-19 differences in individual risk profiles for MRSA acquisition as well as a broad suite of properties of the hospital social network using person-location and person-person interactions inferred from the electronic medical record.

Figure 2. Social Network Construction

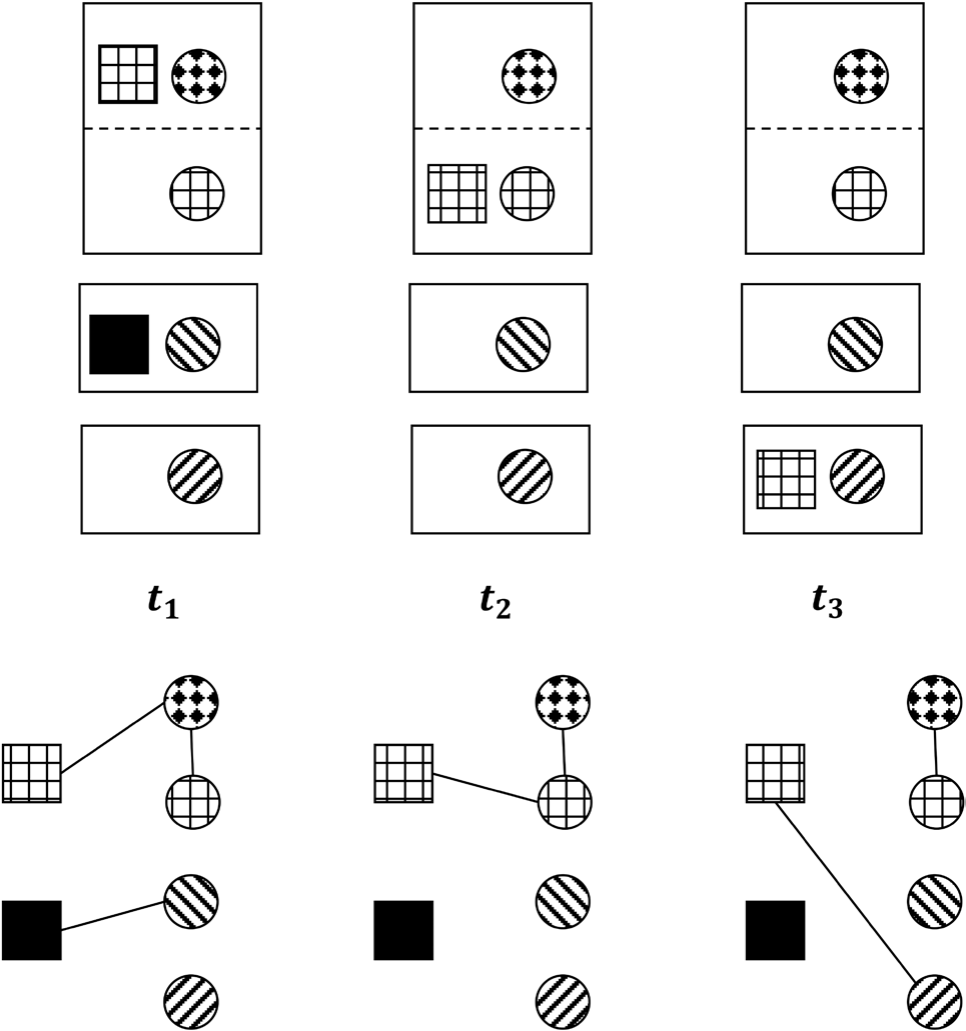

We constructed a contact network of hospitalized patients and staff at University of Virginia Hospital to analyze the properties of both person-location and person-person networks and their changes pre- and post-COVID-19. Colocation data (inferred from shared patient rooms and healthcare personnel (HCP)-patient interactions recorded in the electronic health record, e.g., medication administration) were used to construct contact networks, with nodes representing patients and HCP, and edges representing contacts. The above schematic shows how the temporal networks are inferred. In the figure, circles represent patients and the small filled squares represent HCP, while the larger rectangles represent patient rooms. The first room is a shared room with two patients. At each time step, co-location is inferred from the EMR data, which specifies interactions between HCP and patients. This can be represented as the temporal network (t) at the bottom.

**Results:**

Hand hygiene compliance significantly improved post-COVD-19, in parallel with other infection control measures. Patient Shorr Scores (an index of individual MRSA risk) were statistically similar pre-/post-COVD-19. Analysis of various network properties demonstrated no trends to suggest a reduced outbreak threshold post-COVD-19.

Figure 3. Hand Hygiene Compliance Rates

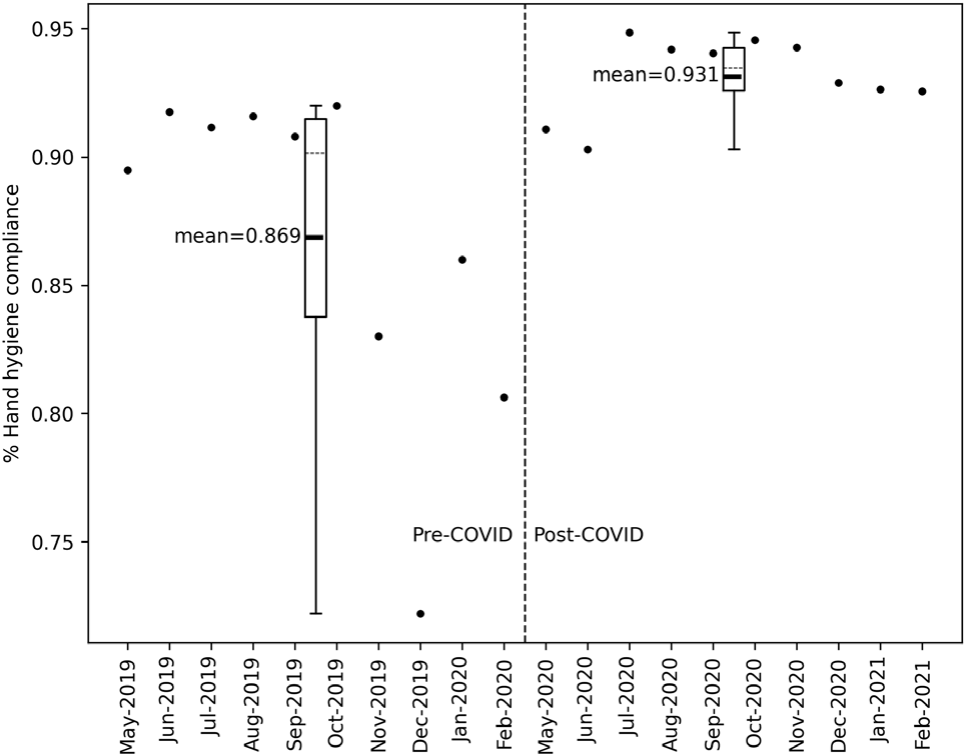

Analysis of hospital-wide hand hygiene auditing data (anonymous auditors deployed to various units across UVA Hospital with an average 1,710 observations per month (range 340 - 7,187)) demonstrated a statistically significant (6%) improvement in average monthly hand hygiene compliance (86.9% pre- versus 93.1% post-COVD-19; P=0.008).

Figure 4. Individual MRSA Risk Factors

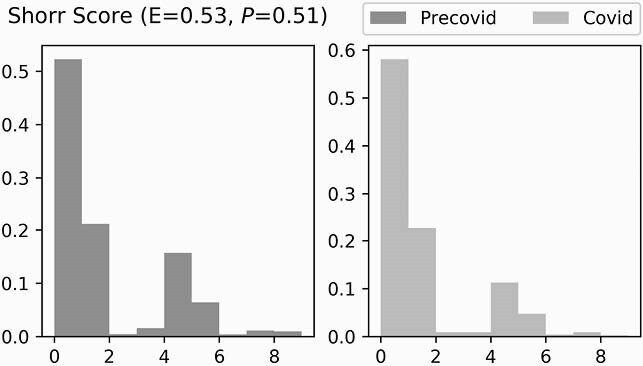

We calculated the Shorr Score (a validated tool to estimate individual risk for MRSA carriage in hospitalized patients; Shorr et al. Arch Intern Med. 2008;168(20):2205-10) for patients using data from the electronic health record to test the hypothesis that individual risk factors in aggregate did not change significantly in the post-COVD-19 period to explain changes in MRSA acquisition. Values for this score ranged from 0 to 10 with the following criteria: recent hospitalization (4), nursing home residence (3), hemodialysis (2), ICU admission (1). Pictured are frequency distributions of Shorr scores in the pre-COVID-19 and post-COVID-19 periods. The Mann-Whitney effect size (E), 0.53 (P=0.51), indicated that pre- and post-COVD-19 distributions were very similar.

Figure 5. Social Network Properties and Analysis (Effect Size, P-Value)

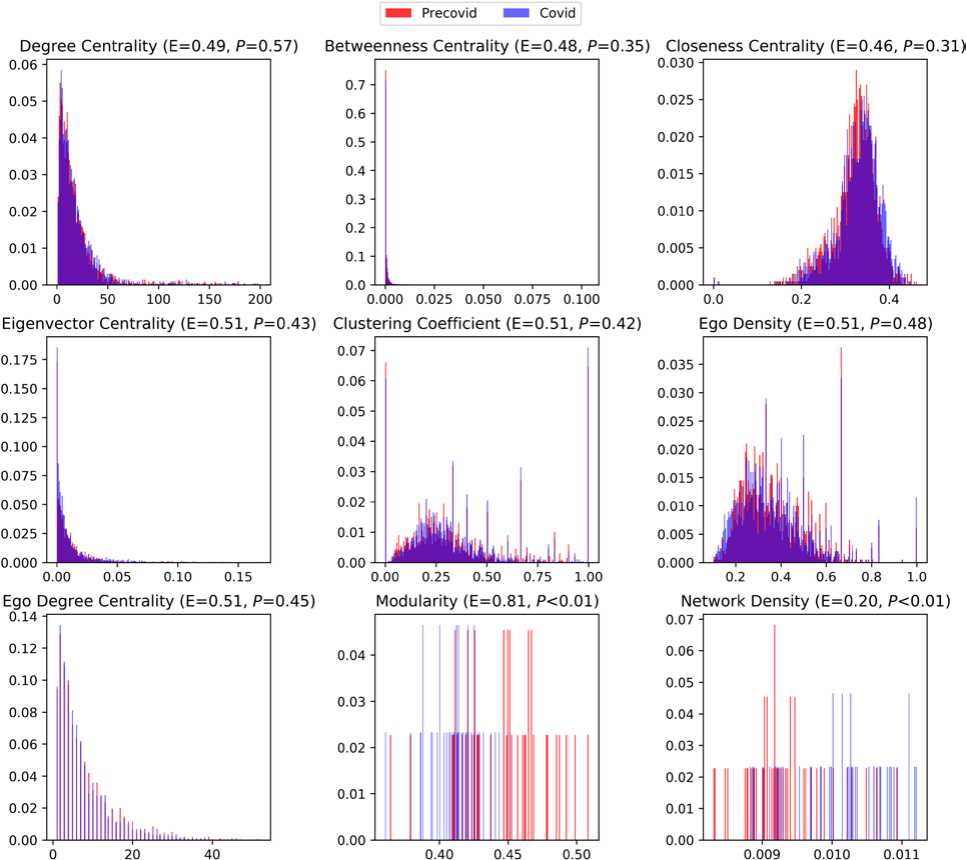

We analyzed three major types of network properties for this analysis: (1) Node properties of the pre- and post-COVID-19 networks consisted of all the edges in the pre- and post-COVID-19 periods, respectively. We considered a number of standard properties used in social network analysis to quantify opportunities for patient-patient transmission: degree centrality (links held by each node), betweenness centrality (times each node acts as the shortest ‘bridge’ between two other nodes), closeness centrality (how close each node is to other nodes in network), Eigenvector centrality (node’s relative influence on the network), and clustering coefficient (degree to which nodes cluster together) in the first five panels (left to right, top to bottom); (Newman, Networks: An Introduction, 2010). Each panel shows the frequency distributions of these properties. These properties generally did not have a normal distribution and therefore we used a Mann Whitney U test on random subsets of nodes in these networks to compare pre- and post-COVID properties. The mean effect size (E) and P-values are shown for each metric in parenthesis. We concluded that all of these pre- versus post-COVID-19 network properties were statistically similar. (2) Properties of the ego networks (networks induced by each node and its ‘one-hop’ neighbors). We considered density (average number of neighbors for each node; higher density generally favors lower outbreak threshold) and degree centrality (number of links held by each node) of ego networks (middle right and bottom left panels). The mean effect size and p-values using the Mann Whitney test are shown in parenthesis; there were no statistically significant differences in these properties in the pre- and post-COVID networks. (3) Aggregate properties of the weekly networks, consisting of all the interactions within a week. We considered modularity (measure of how the community structure differs from a random network; higher modularity means a stronger community structure and lower likelihood of transmission) and density (average number of neighbors each node; higher density generally favors lower outbreak threshold) of the weekly networks (bottom middle and bottom right panels). The modularity in the post-COVID weekly networks was slightly lower (i.e., it has a weaker community structure, and the network is more well mixed), while density was slightly higher, the differences of which were statistically significant; a caveat is that these are relatively small datasets (about 40 weeks). These differences (higher density, and better connectivity) both increase the risk of transmission in the post-COVID networks. In summary, the post-COVID networks either have similar properties as the pre-COVID networks, or had changes which are unlikely to have played a role in reducing MRSA transmission.

**Conclusion:**

A significant reduction in post-COVD-19 MRSA transmission may have been an unintended positive effect of enhanced infection control measures, particularly hand hygiene and increased mask use. A modest (11.6%) post-COVD-19 reduction in surveillance testing may have also played a role. Despite pandemic-related cohorting and census fluctuations, most network properties were not significantly different post-COVID-19, except for aggregate density and modularity which varied in a direction that instead favored transmission; therefore, HCP-based networks did not play a significant role in reducing MRSA transmission. Multivariate modeling to isolate relative contributions of these factors is underway.

Figure 6. Surveillance Testing and Clinical Culturing

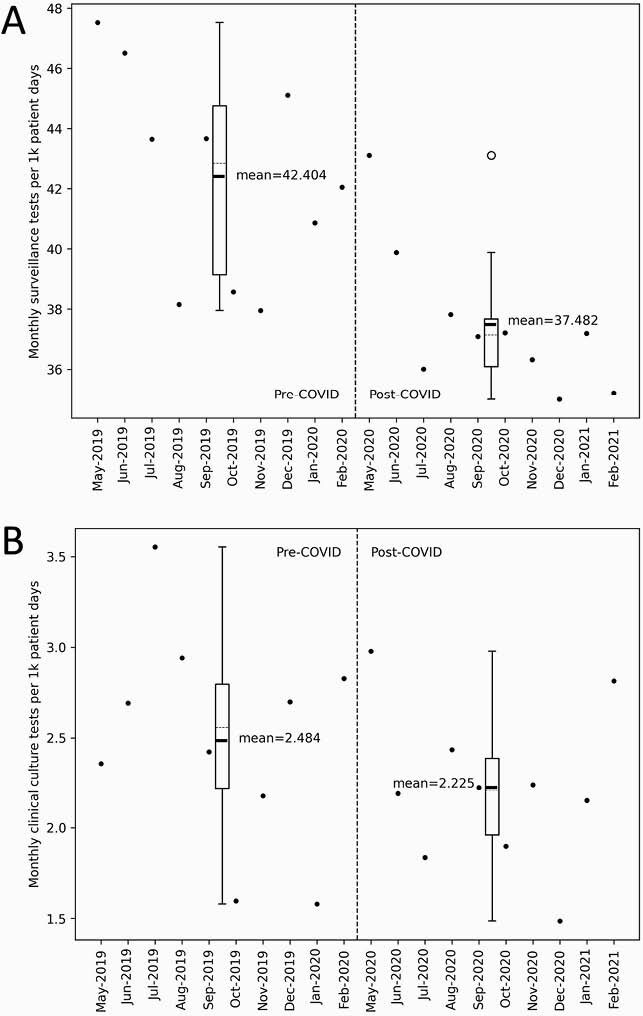

Post-COVD-19, there was a modest (11.6%) but statistically significant reduction in surveillance PCR testing (42.4 mean tests per 1,000 patient days pre- versus 37.5 post-COVD-19; P<0.002). There was not a statistically significant difference in rates of clinical cultures sent (2.48 cultures per 1,000 patient days pre- versus 2.23 post-COVD-19; P=0.288).

**Disclosures:**

**All Authors**: No reported disclosures

